# Effects of High-Intensity Interval Training Protocols on Liver Enzymes and Wellness in Women

**DOI:** 10.1155/2021/5554597

**Published:** 2021-04-30

**Authors:** Timothy A. Rengers, Samantha C. Orr, Charles R. C. Marks, Tamara Hew-Butler, Myung D. Choi, Scotty J. Butcher, Dorin Drignei, Elise C. Brown

**Affiliations:** ^1^Department of Interdisciplinary Health Sciences, Oakland University, 433 Meadowbrook Road, Rochester 48309, MI, USA; ^2^Department of Human Movement Sciences, Oakland University, 433 Meadowbrook Road, Rochester 48309, MI, USA; ^3^College of Education, Wayne State University, Detroit, MI, USA; ^4^School of Rehabilitation Science, College of Medicine, University of Saskatchewan, 104 Clinic Place, Saskatoon, Saskatchewan, Canada S7N 2Z4; ^5^Department of Mathematics and Statistics, Oakland University, 146 Library Drive, Rochester 48309, MI, USA; ^6^Department of Public and Environmental Wellness, Oakland University, 433 Meadowbrook Road, Rochester 48309, MI, USA

## Abstract

**Background:**

Single-modality, high-intensity interval training (HIIT) using traditional cardiorespiratory exercise selection has been found to provide similar and sometimes superior cardiometabolic effects compared with moderate-intensity continuous training. However, little is known regarding the cardiometabolic and psychosocial effects of HIIT using resistance training modalities. Therefore, this study aims to compare the effects of HIIT using rowing (R-HIIT) and multimodal HIIT (MM-HIIT) using resistance training on liver enzymes, cardiometabolic risk factors, and psychosocial outcomes.

**Method:**

Recreationally active females with a body mass index <30 kg/m^2^ (*N* = 16, 23.0 ± 5.9 years) were randomized into a MM-HIIT or R-HIIT group and completed a 12-week HIIT intervention (ClinicalTrials.gov registration number: https://clinicaltrials.gov/ct2/show/NCT03093441) using principles of social cognitive theory (SCT). Participants completed pre- and postintervention measurements on anthropometrics, resting heart rate, blood pressure, blood measures (lipids, liver enzymes, and glucose), exercise self-efficacy, and perceived wellness. Analysis of covariance was used to examine differences in postintervention measures between groups after controlling for baseline values, waist circumference, and waist-to-height ratio.

**Results:**

R-HIIT group had significantly decreased alanine aminotransferase (mean difference = 13.16, *P*=0.013, effect size (ES) = 0.44, confidence interval (CI) = 3.40 to 22.92) and aspartate aminotransferase (mean difference = 10.79, *P*=0.024, ES = 0.38, CI = 1.67 to 19.90) levels compared with the M-HIIT group, and the whole group had improved wellness scores (14.72 ± 2.6 to 16.89 ± 2.76, *P*=0.002).

**Conclusion:**

R-HIIT may be an effective preventative method for improving liver health in females without obesity. When using principles of SCT, HIIT may enhance overall well-being.

## 1. Introduction

Cardiometabolic diseases, such as metabolic syndrome and type 2 diabetes, currently affect 23% and 13.2% of the US adult population, respectively, and the prevalence estimates increase with age [1]. These conditions are closely linked to lifestyle factors, such as exercise, and lead to increased mortality rates, especially when cardiometabolic multimorbidity is present [2]. These trends highlight the need for early prevention efforts in young adults.

Traditional risk factors for cardiometabolic diseases include blood lipids, blood pressure, glucose, and abdominal adiposity [1]. Since the liver plays an important role in the development of cardiometabolic disease, as a paramount location for glucose metabolism, cholesterol synthesis, and fatty acid oxidation, more novel risk factors include liver enzymes, alanine aminotransferase (ALT), and aspartate aminotransferase (AST) [3]. These enzymes are markers of liver damage linked to excessive hepatic fat accumulation and are used as surrogate markers for detecting nonalcoholic fatty liver disease [4]. Additionally, the prevalence of cardiometabolic risk factors tends to be higher in women compared with men, and, subsequently, women have higher prevalence rates of metabolic syndrome [5].

Exercise using high-intensity interval training (HIIT) has been found to be an effective means of reducing cardiometabolic risk, specifically, diastolic blood pressure (DBP) and fasting glucose, in populations that are overweight or obese, but few studies have investigated these effects in normal weight populations [6]. Exercise using resistance training, however, can reduce systolic blood pressure (SBP), DBP, mean arterial pressure, resting heart rate (RHR), total cholesterol (TC), triglycerides, fasting insulin, fasting glucose, and C reactive protein and increase high-density lipoprotein cholesterol (HDL-c), with greater effects in older compared with younger adults [7]. While most HIIT research has focused on the use of training modalities commonly used to improve cardiorespiratory fitness (e.g., running and cycling) in exercise protocols [6], another form of HIIT, multimodal HIIT (MM-HIIT), has become a popular training method in fitness gyms worldwide [8]. Recently, it has been demonstrated that MM-HIIT, which utilizes resistance training and functional movements, can produce similar aerobic adaptations compared with HIIT using rowing but with greater muscle performance, including strength, in females [9]. Given that resistance training can improve a range of cardiometabolic markers in healthy adults [7], it is important to determine if MM-HIIT has an influence on these risk profiles.

The effects of exercise on ALT and AST are less clear in healthy adults. While both aerobic and resistance exercise can decrease ALT in patients with nonalcoholic fatty liver disease [10], a study by Moro et al. [11] found no change in ALT or AST in healthy young adults following interventions involving high-intensity interval resistance training or resistance training. Furthermore, a study by Skrypnik et al. [12] found that the combination of resistance and endurance training was more effective than endurance training alone in improving liver function in females with abdominal obesity. Understanding the effects of exercise on liver enzymes in young adults is important for disease prevention.

A criticism of HIIT is that it may be too strenuous and poorly tolerated by sedentary populations [13], and some have argued that the general public may not have the confidence to engage in such challenging protocols [14]. This is problematic since self-efficacy is one of the most consistent predictors of physical activity in adults [15], and exercise intensity preference and tolerance are known to be strong indicators of exercise participation [16]. However, single-bout HIIT studies have found that adults reported higher levels of self-efficacy when the duration of HIIT bouts was shortened and the number of repetitions was increased [17] and similar levels of self-efficacy when engaging in HIIT compared with moderate-intensity continuous exercise [18].

Social cognitive theory (SCT) has been used to understand and predict a wide variety of physical activity and/or exercise behaviors and also design interventions that promote physical activity behaviors [19]. This theory assumes that there is a dynamic relationship between individual factors, environmental factors, and behavior [20]. Exercise participation and adherence, therefore, are thought to be partially influenced by self-efficacy, and one's confidence in their ability to be active is determined by factors such as mastery experience, social modeling, improved physical and emotional states, and verbal persuasion. It has been found that verbal encouragement during HIIT bouts leads to greater participant satisfaction than no encouragement and attenuated a decline in self-efficacy [21]. It has also been demonstrated that self-efficacy positively predicts perceived wellness, which suggests that an increase in self-efficacy may influence well-being in a beneficial manner [22].

Therefore, the purpose of this study was to (1) determine differences in liver enzymes and cardiometabolic outcomes and (2) psychosocial outcomes using principles of SCT between MM-HIIT and aerobic-style rowing HIIT (R-HIIT) in healthy females after a 12-week intervention. A secondary aim was to determine within-group changes in cardiometabolic and psychosocial outcomes. The hypotheses were as follows: (1) MM-HIIT will yield superior liver enzyme and cardiometabolic effects compared with R-HIIT and (2) HIIT will induce similar increases in exercise self-efficacy and perceived wellness in both groups.

## 2. Materials and Methods

### 2.1. Study Population

Following approval from the Oakland University Institutional Review Board on April 6, 2017 (reference number: 1002886-3), participant recruitment was conducted at a US university from April 2017 to July 2017. Eligibility criteria included recreationally active females without obesity (body mass index < 30 kg/m^2^) aged 18–40 years who do not engage in a regular training program. Recreationally active was defined as periodically participating in physical activity or exercise between one and three hours a week for at least a month but not involved in a systematic endurance or weight training activity. Participants were excluded if they had a history of fainting when having their blood taken, exercise-limiting cardiovascular, respiratory, metabolic, or musculoskeletal illness/injury, or if they were currently taking medication that would alter the physiological responses to exercise.

Participants were recruited through email, flyers, banners, recruitment tables, and class announcements. Interested participants contacted the primary investigator, and screening interviews were scheduled. Written informed consent was obtained prior to study enrollment, and appropriate standards for human experimentation in accordance with the Helsinki Declaration were followed [23]. A total of 28 recreationally active females were initially recruited. Following the medical clearance, four were not eligible to participate in the study, four decided not to participate, and two did not respond to follow-up contact efforts. A more detailed flow of participants throughout the study can be found elsewhere.^9^

### 2.2. Study Design

A 12-week parallel-group randomized trial was conducted at the university's recreation center. Eighteen participants were evenly randomized into either the MM-HIIT or the R-HIIT group using a computerized random number generator. The intervention was initially designed with a control group, but the sample produced from participant recruitment efforts was significantly smaller than predicted. Student researchers (undergraduate and graduate) led the intervention in a group setting, under the supervision of the principal investigator. Measurements were taken at the university by researchers and trained student researchers, and assessors were not blinded to group assignment. All participants had measures of height, mass, WC, RHR, blood pressure, biomarkers (glucose, electrolytes, triglyceride (TG), TC, high-density lipoprotein cholesterol (HDL-C), low-density lipoprotein cholesterol (LDL-C), AST, and ALT), exercise intensity preference and tolerance, exercise self-efficacy, and perceived wellness assessed at baseline and after the intervention.

### 2.3. Theoretical Framework

Principles of SCT were employed in order to promote continued exercise after the intervention, and these included mastery experience, social modeling, improving physical and emotional states, and verbal persuasion [20]. Regarding the mastery experience, all participants performed the same specific warm-up protocol throughout the intervention, regardless of which group they were assigned to, in order to develop mastery of the movements, so that they would develop self-efficacy in participating in resistance training even after the twelve weeks have passed. Social modeling was employed such that either the principal investigator or a research assistant would demonstrate each of the movements and provide points of performance for the participants to focus on while executing the respective movement. Verbal persuasion was used as the researcher personnel not only offered feedback and cueing throughout the sessions but also provided words of encouragement. Each participant received the same quality and quantity of encouragement throughout the intervention and during the muscular and aerobic fitness testing.

Outcome expectations of participating in HIIT were predicted to improve as physical and emotional states were likely to be impacted through improving fitness and health by participating in the HIIT intervention and providing a positive, supportive environment that was created by the research personnel. Three different psychosocial surveys were administered before and after the intervention to determine the efficacy of the theoretical framework.

## 3. Measurements

### 3.1. Body Composition

Height and body mass were measured with shoes and extra clothing removed using a mechanical stadiometer and scale, respectively. Body mass index (BMI) was calculated as mass (kg)/height^2^ (m). Waist circumference (WC) was assessed on the right side of the body on the superior lateral border of the pelvis at the intersection of the midaxillary line using a tape measure (Gulick II; Country Technology, Inc., Gays Mills, WI). Waist-to-height ratio (WHtR) was calculated as WC (cm)/height (cm). Other body composition variables (lean mass, bone density, and fat mass) were assessed using dual-energy X-ray absorptiometry and have been reported elsewhere [9].

### 3.2. Heart Rate and Blood Pressure

Prior to venipuncture, RHR and blood pressure were measured using an Accutorr 3 spot check vital sign monitor (Mindray North America, Mahwah, NJ) and taken in a seated position from the left arm after the participant rested for ten minutes. Blood pressure was taken in triplicate with 3 min in between each reading, and the average of the second and third measurements was used.

### 3.3. Blood Measures

Following a 12-hour fast, participants completed both pre- and postintervention venipuncture performed by a trained phlebotomist. Before sampling, participants were asked by a researcher and the phlebotomist if they had anything to eat or drink that morning. Venous samples were taken between 6 am and 10 am, and breakfast was provided afterwards. Samples were collected in a lithium heparin BD Vacutainer Plasma Tube and immediately placed on ice after collection. Whole blood was analyzed within 30 minutes of collection using Abaxis Piccolo Xpress point of care chemistry analyzer (Abaxis, Inc, Union City, CA) and Lipid Panel Plus discs assays for the measurement of TC, HDL-C, TG, ALT, AST, glucose, and LDL-C. Prior to blood analysis, calibration and quality control measures were taken by running control reagents, and all values fell within the acceptable range. Although Batacan and colleagues [6] found no significant changes in lipid profile with individuals with normal lipids, much of the existing literature pertains to participants with abnormal blood lipids. Lipids have been included in this study as they are criteria required to assess cardiometabolic risk factors.

### 3.4. Exercise Self-Efficacy

The 18-item Exercise Self-efficacy (ESE) Scale [24] assesses one's ability to regulate their exercise adherence on a regular basis. This scale has been shown to be an acceptable measure in Australian cardiac rehabilitation patients [25]. Psychometric properties of this tool include a high internal consistency of 0.95, high intercorrelation coefficient of 0.90 across items, single-factor structure that explained 58% of the variance in scores, and an absence of floor or ceiling effects. Additionally, sufficient concurrent validity was demonstrated as those who walked greater distances in the 6-minute walk test had significantly higher ESE scores compared with those who walked shorter distances (*P*=0.044), and sensitivity to change over time was evident as the change in ESE scores positively correlated with distance travelled on the 6-minute walk test (*r* = 0.28; *P*=0.035). This scale asked participants how likely they thought that they would exercise on a regular basis under a range of different circumstances, such as when they felt depressed, after a vacation, or without support from family or friends. The scale was administered before and after the intervention. Participants would rate their confidence in their likelihood to exercise for each circumstance on a scale of 0 (cannot do at all) to 100 (highly certain can do).

### 3.5. Perceived Wellness

The 36-item Perceived Wellness Survey [26] measures one's perception of their own personal health across dimensions of physical, spiritual, psychological, social, emotional, and intellectual wellness. This measure has been demonstrated as a useful tool in university students [27]. Internal consistency of the scale ranged from 0.88 to 0.93, discriminant validity was evident as health professionals were able to identify participants with low and high degrees of wellness (*t* = 5.46, *P* ≤ 0.05, df = 38), and a single-factor structure that explained 24% of the variance was observed. This survey asked the participants to rate on a scale of 1 (very strongly disagree) to 6 (very strongly agree) their personal health perceptions and was administered before and after the intervention. An example of one of the items related to physical wellness is “my physical health has restricted me in the past.”

### 3.6. Exercise Intensity Preference and Tolerance

The Preference for and Tolerance of the Intensity of Exercise Questionnaire consists of two eight-item scales, and was administered before and after the intervention. This questionnaire was used to measure individual differences in both the preference for and tolerance of exercise intensity [28]. This questionnaire has demonstrated acceptable validity and reliability in university students [29]. This tool asks participants to indicate using a scale of 1 (I totally disagree) to 5 (I totally agree) whether or not they agree with statements related to their own exercise intensity preferences, such as “when I exercise, I usually prefer a slow, steady pace,” and tolerance, such as “while exercising, I try to keep going even after I feel exhausted.”

### 3.7. Exercise Intervention

Details of the intervention have been described elsewhere [9]. Briefly, both groups completed three 60-minute sessions each week for 12 weeks (warm-up and instruction, HIIT session, and cool-down) in a group setting. Each HIIT session comprised 60 seconds of maximum intensity work followed by three minutes of rest for a total of six sets. The MM-HIIT group's work bout comprised four to six repetitions of a heavy barbell movement (e.g., back squat and bench press) and eight to ten repetitions of moderate-intensity assistance exercise (e.g., dumbbell lunges and bent-over rows), and for the remainder of the 60 seconds, they would complete as many repetitions as possible of a conditioning modality (e.g., jump rope and ball slams). Eight different workouts were rotated through during the 12 weeks in a recurring pattern, and intensity or speed was increased each time a repeated workout was scheduled. The R-HIIT group rowed for the entire 60 seconds and would aim to increase the number of meters rowed each bout. The R-HIIT group used a rowing ergometer (Concept2, Morrisville, VT).

Participants wore a heart rate monitor (Polar FT1, Bethpage, NY) in order to monitor intensity (>90% of age estimated maximum heart rate), and their rate of perceived exertion was recorded using Borg's modified 10-point scale [30]. Prior to starting the intervention, participants were instructed to maintain throughout the intervention period the same eating and physical activity habits they had prior to the start of the study. Physical activity was monitored weekly using Godin's Leisure Time Activity Questionnaire [31].

### 3.8. Statistical Analyses

Independent samples *t* tests were used to determine differences between the R-HIIT and MM-HIIT groups at baseline, differences between groups in percentage of predicted maximum heart rate (220–age) maintained during HIIT, and differences in exercise intensity tolerance and preference between those who dropped out of the study and those who completed the intervention. An analysis of covariance (ANCOVA) was computed to compare postintervention means after controlling for baseline values with the Bonferroni post-hoc comparison to determine significant treatment differences for all outcomes. Given that higher WC and WHtR have been linked to increased cardiometabolic risk [32], WC and WHtR were included as covariates in the ANCOVA used for cardiometabolic risk variables. Effects sizes were computed using partial eta squared (*η*p^2^) (0.01 = small effect; 0.06 = moderate effect; 0.14 = strong effect). Paired samples *t* tests were used to examine within-group changes before and after the intervention for outcome variables. Statistical analyses were computed in SPSS Version 25. Statistical procedures were performed in SAS v9.4. Significance level was set a priori at 0.05. Power calculations and sample size estimates have been reported elsewhere, and sample size was calculated using G ∗ Power 3.1.9.2 [9]. Briefly, a total of 12 participants were necessary to detect changes in the primary variable of interest for the original project, VO2max, based on a power of 0.80 and *α*-level of 0.05. For the present study, a post-hoc power analysis was conducted, and a power of 0.90 was achieved respective to changes in ALT.

## 4. Results

Significant differences were found between groups at baseline for TC (*P*=0.002) and LDL-C (*P*=0.001). Groups were similar for all other outcomes.  Table 1 describes participant characteristics at baseline. Of the 18 participants who started the intervention, two from the R-HIIT group dropped out of the study due to an unexpected commitment and a concussion that occurred for reasons outside of the study. Exercise intensity tolerance and preference were similar between those who dropped out of the study and those who completed it. Therefore, 16 participants completed the intervention and were included in the analyses. Adherence-to-session-attendance rates were similar between the MM-HIIT (84% of sessions) and R-HIIT (81% of sessions). During the exercise sessions, the mean estimated percentage of maximum heart rate was similar for both groups (*P*=0.50, R-HIIT = 90.8% and MM-HIIT = 89.6%). Physical activity outside of the intervention from week one to week 12 did not change for the MM-HIIT (*P*=0.79) or R-HIIT (*P*=0.87).

### 4.1. Body Composition

No changes in BMI, WC, or WtHR occurred, and other body composition variables have been reported elsewhere [9].

### 4.2. Heart Rate and Blood Pressure

No significant changes occurred for RHR or blood pressure.  Table 2 presents the ANCOVA results for cardiometabolic risk variables.

### 4.3. Blood Measures

**Figure 1:**
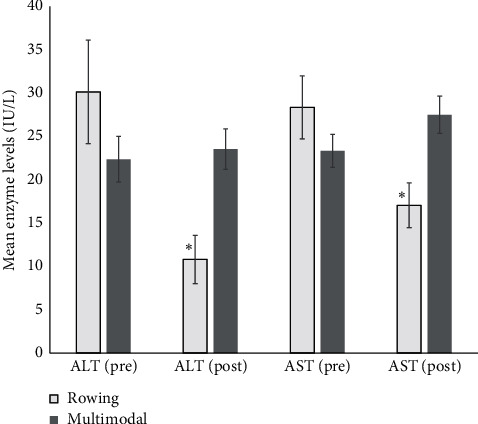
Unadjusted preintervention and adjusted postintervention ALT and AST mean values in rowing and multimodal groups with standard error. ^*∗*^Significant change from before to after the intervention. ALT: alanine aminotransferase. AST: aspartate aminotransferase.

The ANCOVA revealed a significant decrease in ALT (*P*=0.01) and AST (*P*=0.02) in the R-HIIT group compared with the MM-HIIT group, and the results are presented in Table 2. Paired *t* tests showed a significant decrease in ALT for the R-HIIT group (*P*=0.02). Changes in ALT and AST for each group are presented in Figure 1. No other significant changes were observed.

### 4.4. Psychosocial

For psychosocial variables, the ANCOVA showed no between-group differences and paired *t* tests revealed no change in the MM-HIIT or R-HIIT group for any of the psychosocial variables. The results are presented in Table 3. For the group as a whole, *t* tests showed a significant increase in perceived wellness (mean = 14.72 ± SD = 2.6 to 16.89 ± 2.76, *P*=0.002), and a significant increase in wellness was observed in the MM-HIIT group (14.70 ± 2.89 to 16.73 ± 3.08, *P*=0.002). No other significant findings were present.

## 5. Discussion

This was a 12-week intervention study that examined the effects of MM-HIIT and R-HIIT on liver enzymes, cardiometabolic risk factors, and psychosocial outcomes in healthy young females without obesity. To the authors' knowledge, this was the first study to investigate the impact of MM-HIIT on these specified outcomes. The results were contrary to the author's hypothesis and showed that R-HIIT was more effective in improving liver enzymes compared with MM-HIIT. HIIT, when using principles of SCT, may result in improved well-being regardless of modality. No significant changes were observed in any of the cardiometabolic risk variables, which was likely due to the participants already being in a healthy state with little room for improvement. This was consistent with a meta-analysis by Batacan et al. [6], which showed that HIIT improved VO2max in normal weight populations, with no effect on BMI, % body fat, resting HR, SBP, or DBP. Further studies, in particular with a control group and increased sample size, are required to increase the reliability of these results.

Liver enzymes, ALT and AST, were effectively reduced in the R-HIIT group and not the MM-HIIT group after controlling for baseline values, WC, and WHtR. These findings were consistent with other HIIT studies of a similar duration [33, 34]. Hallsworth and colleagues [34] demonstrated a reduction in intrahepatic lipid with a concomitant decrease in ALT and AST after 12 weeks of cycling HIIT in individuals with nonalcoholic fatty liver disease. The HIIT protocol included a two-minute work period with 10 seconds added every week and a three-minute recovery period comprised of 90 seconds of rest, 60 seconds of light resistance training using bands, and 30 seconds for transitioning. Another study that used the same HIIT protocol as Hallsworth and colleagues [34] investigated the effects of HIIT in patients with type 2 diabetes [33]. Similarly, in the HIIT group, they found a significant decrease in liver fat compared with controls and a within-group decrease in ALT and AST, which not significantly different from controls. It may be that the R-HIIT group experienced a reduction in intrahepatic lipids, which led to a reduction in ALT and AST. While some have suggested that aerobic training is more effective than resistance training in decreasing liver fat due to differences in caloric expenditure between the modalities [35], others have found that, with sufficient training volume and intervention duration, resistance training was similarly as effective as aerobic training in reducing hepatic fat [36]. Although no change was observed in blood lipid levels in the R-HIIT group, intrahepatic lipid may still have been reduced by modifications to the oxidation or secretion of intrahepatic lipids [37]. For example, aerobic exercise studies in rats have found that treadmill [38] and wheel running [39] resulted in decreased intrahepatic lipids coinciding with a decrease in enzymes that are involved in hepatic de novo lipogenesis. Resistance exercise has also been shown to beneficially influence intrahepatic lipids, but the suggested mechanisms were related to increased uptake of nonesterified fatty acid in the skeletal muscle, which is thought to lower uptake in the liver [37]. Thus, the mechanisms related to reduction in intrahepatic lipids may vary based on the exercise mode, and further study is needed to understand why R-HIIT may be more effective than MM-HIIT in reducing ALT and AST. Alternatively, the effectiveness of R-HIIT compared with MM-HIIT in improving liver enzymes in the present study may have been related to the significantly higher levels of fasting TC and LDL-C in the R-HIIT group compared with the MM-HIIT group at baseline, since dyslipidemia is linked to increased hepatic fat [40]. Additionally, the baseline mean ALT level for the R-HIIT group was 31.14 ± 16.28 U/L, a value greater than what is considered a healthy range based on clinical guidelines [3].

A novel aspect of the present study was the use of resistance training, including barbells and dumbbells, combined with conditioning modalities in a HIIT protocol (MM-HIIT group). Few studies have investigated the effects of MM-HIIT [41–45], and only one other study used heavy barbell resistance exercises [45]. Given that barbells allow for a greater absolute load to be moved through space compared with other forms of resistance training implements (e.g., resistance bands, one's own body weight, and dumbbells), the higher training loads when using barbells have the potential to result in greater cardiometabolic improvements [46]. Future work should investigate the effects of MM-HIIT using heavy barbell exercises in populations with increased cardiometabolic risk profiles.

The hypothesis that participating in a HIIT intervention that used SCT principles would increase perceived wellness as a result of increased exercise self-efficacy was not supported. While a significant increase in perceived wellness occurred in the group as whole, no changes were observed in exercise self-efficacy. The lack of improvement in self-efficacy may have been due to the use of recreationally active participants, who were primarily affiliates of the School of Health Sciences and may have already had high enough levels of confidence in terms of exercising on a regular basis. There is currently a lack of evidence that demonstrates that HIIT interventions can result in improved psychological well-being [47]. While no group differences were observed, the findings suggested that perceived wellness increased in the whole group before and after the intervention. Given that both groups completed the intervention together in the same setting, they were exposed to similar social environmental effects. These findings suggest that participating in HIIT may positively influence social cognitions. Participation in HIIT in a group setting with an emphasis on developing mastery of exercise movements, use of competent role models, and providing a positive, supportive environment through verbal encouragement may increase well-being in females.

Limitations of this study included the lack of a control group and small sample size, and the results should be interpreted with caution. Inclusion of a control group in future work would increase the likelihood that any changes in outcomes were the result of the intervention and not factors outside of the study. Although the sample size was sufficient for detecting changes in ALT, more participants may have been necessary for analyzing change in cardiometabolic and psychosocial variables. Both of these limitations were related to limited resources. Use of multiple intervention sites and recruiting outside of the campus community in future investigations would allow for a larger sample size and the inclusion of a control group. Another limitation is the health status of the participants, which may have hindered improvement in cardiometabolic risk variables since most of the baseline means were in a healthy range. Additionally, groups were not matched for energy expended. Moreover, it is unclear whether the warm-up contributed to the results or not. Further investigations should compare these protocols in those with increased cardiometabolic risk and ensure that the sample size is sufficient to detect changes in cardiometabolic and psychosocial outcomes.

## 6. Conclusion

R-HIIT may be useful for improving liver metabolic health, even in those who are not obese. Cardiometabolic parameters did not change in the MM-HIIT group possibly due to the health status of the participants, and future work should investigate the effects of MM-HIIT using heavy barbell exercises in populations with increased cardiometabolic risk, who may be more physiologically amenable to change. When HIIT is conducted in a group setting, which is popular in many health, fitness, and sport performance facilities worldwide, with the use of SCT principles, it has the potential to improve well-being. It is important to focus on the enhancement of psychological health alongside cardiometabolic health for better exercise adherence after the intervention is complete, which will ultimately enhance long-term health outcomes and reduce mortality [47].

## Figures and Tables

**Table 1:** Participant descriptive statistics between groups at baseline.

	MM-HIIT group	R-HIIT group	*P* value
Number of participants (n)	9	7	
Age (years)	23.78 ± 6.40	22.00 ± 2.83	0.507
Height (cm)	167.31 ± 6.65	156.54 ± 4.87	^*∗*^0.003
Body mass (kg)	64.60 ± 9.83	60.39 ± 5.77	0.333
BMI (kg/m^2^)	23.02 ± 2.69	24.68 ± 2.38	0.220
WC (cm)	85.06 ± 5.81	83.70 ± 6.25	0.660
Resting heart rate (bpm)	68.56 ± 4.95	74.71 ± 8.75	0.096
Systolic blood pressure (mm/Hg)	109.78 ± 4.33	113.14 ± 9.07	0.342
Diastolic blood pressure (mm/Hg)	70.61 ± 6.01	75.00 ± 5.98	0.169
Triglycerides (mg/dL)	96.67 ± 60.83	83.86 ± 19.62	0.603
Glucose (mg/dL)	91.89 ± 7.72	95.14 ± 7.38	0.408
Total cholesterol (mg/dL)	150.78 ± 20.14	189.71 ± 20.45	^*∗*^0.002
HDL-c (mg/dL)	57.11 ± 11.17	60.86 ± 5.18	0.428
LDL-c (mg/dL)	74.67 ± 15.85	111.71 ± 16.59	^*∗*^0.001
ALT (U/L)	23.11 ± 8.21	31.14 ± 16.28	0.217
AST (U/L)	24.11 ± 5.88	29.29 ± 9.93	0.213
Weekly leisure physical activity score	32.67 ± 17.85	23.00 ± 14.24	0.262
Exercise self-efficacy	63.09 ± 17.88	65.95 ± 23.42	0.785
Perceived wellness	14.70 ± 2.89	14.75 ± 2.40	0.968
Exercise intensity tolerance	28.22 ± 3.73	23.14 ± 5.40	0.059
Exercise intensity preference	27.00 ± 5.10	29.00 ± 5.23	0.454

All results are presented as means and standard deviations except for number of participants which is presented as *n*. ^*∗*^Significant differences at the *P* < 0.05 level. WC = waist circumference; HDL-c = high-density lipoprotein; LDL-c = low-density lipoprotein; ALT = alanine aminotransferase; AST = aspartate aminotransferase; MM-HIIT = multimodal high-intensity interval training; R-HIIT = rowing high-intensity interval training.

**Table 2:** Summary of changes in cardiometabolic variables over time between R-HIIT (*n* = 7) and MM-HIIT (*n* = 9) groups from ANCOVA.

Outcome and group	Baseline (unadjusted means)	Postintervention (unadjusted means)	Postintervention (adjusted means)	Adjusted mean difference (95% CI)	Effect size (partial eta squared)	*P* value
*Resting heart rate* (bpm)						
R-HIIT	74.71 ± 8.75	74.14 ± 5.84	70.60 ± 8.09	−2.17 (−12.45 to 8.11)	0.019	0.651
MM-HIIT	68.56 ± 4.95	67.22 ± 6.63	68.43 ± 7.72			

*Systolic blood pressure* (mm/Hg)						
R-HIIT	113.14 ± 9.07	112.90 ± 6.34	114.00 ± 4.75	−3.26 (−9.24 to 2.72)	0.116	0.255
MM-HIIT	109.78 ± 4.33	111.59 ± 5.19	110.74 ± 4.54			

*Diastolic blood pressure* (mm/Hg)						
R-HIIT	75.00 ± 5.98	74.10 ± 5.44	73.72 ± 4.43	−3.273 (−8.93 to 2.38)	0.129	0.229
MM-HIIT	70.61 ± 6.01	70.15 ± 5.30	70.44 ± 4.22			

*Triglycerides* (mg/dL)						
R-HIIT	83.86 ± 19.62	82.43 ± 45.90	78.92 ± 46.01	30.70 (−27.69 to 89.09)	0.109	0.272
MM-HIIT	96.67 ± 60.83	106.89 ± 62.93	109.62 ± 43.92			

*Glucose* (mg/dL)						
R-HIIT	95.14 ± 7.38	96.71 ± 8.18	94.04 ± 5.88	2.05 (−5.33 to 9.42)	0.033	0.553
MM-HIIT	91.89 ± 7.72	94.00 ± 7.00	96.08 ± 5.64			

*Total cholesterol* (mg/dL)						
R-HIIT	189.71 ± 20.45	182.29 ± 19.54	168.96 ± 20.72	−12.04 (−38.92 to 14.84)	0.080	0.346
MM-HIIT	150.78 ± 20.14	146.56 ± 16.37	156.92 ± 19.60			

*HDL-c* (mg/dL)						
R-HIIT	60.86 ± 5.18	56.57 ± 7.70	55.76 ± 7.13	−0.14 (−9.16 to 8.89)	0.000	0.974
MM-HIIT	57.11 ± 11.17	55.00 ± 13.24	55.63 ± 6.82			

*LDL-c* (mg/dL)						
R-HIIT	111.71 ± 16.59	109.00 ± 12.57	96.80 ± 17.72	−17.31 (−40.79 to 6.16)	0.193	0.133
MM-HIIT	74.67 ± 15.85	70.00 ± 16.24	79.49 ± 16.60			

*ALT* (U/L)						
R-HIIT	31.14 ± 16.28	16.43 ± 6.32	11.16 ± 7.63	13.16 (3.40 to 22.92)	0.444	^*∗*^0.013
MM-HIIT	23.11 ± 8.21	20.22 ± 7.84	24.32 ± 7.26			

*AST* (U/L)						
R-HIIT	29.29 ± 9.93	22.29 ± 2.75	17.62 ± 7.05	10.79 (1.67 to 19.90)	0.381	^*∗*^0.024
MM-HIIT	24.11 ± 5.88	24.78 ± 7.45	28.41 ± 6.68			

Plus-minus values are means ± SD with postintervention means being adjusted for covariates of baseline values, waist circumference, and waist-to-height ratio, and data in parentheses are 95% CI. Mean difference is based on rowing group minus multimodal group after the intervention. HDL-c = high-density lipoprotein; LDL-c = low-density lipoprotein; ALT = alanine aminotransferase; AST = aspartate aminotransferase; MM-HIIT = multimodal high-intensity interval training; R-HIIT = rowing high-intensity interval training.

**Table 3:** Summary of changes in psychosocial variables over time between R-HIIT (*n* = 7) and MM-HIIT (*n* = 9) groups from ANCOVA.

Outcome and group	Baseline (unadjusted means)	Postintervention (unadjusted means)	Postintervention (adjusted means)	Adjusted mean difference (95% CI)	Effect size (partial eta squared)	*P* value
*Exercise self-efficacy*						
MM-HIIT	63.09 ± 17.88	66.67 ± 13.57	67.22 ± 12.17	6.75 (−6.52 to 20.02)	0.09	0.292
R-HIIT	65.95 ± 23.42	61.19 ± 16.36	60.47 ± 12.18			

*Perceived wellness*						
MM-HIIT	14.70 ± 2.89	16.73 ± 3.08	16.75 ± 2.31	−0.335 (−2.852 to 2.182)	0.006	0.778
R-HIIT	14.75 ± 2.40	17.10 ± 2.50	17.08 ± 2.62			

Plus-minus values are means ± SD with postintervention means being adjusted for covariates of baseline values, and data in parentheses are 95% CI. Mean difference is based on rowing group minus multimodal group after the intervention. MM-HIIT = multimodal high-intensity interval training; R-HIIT = rowing high-intensity interval training.

## Data Availability

The data used to support the findings of this study are available from the corresponding author upon request.
